# Identification of key genes controlling breast cancer stem cell characteristics via stemness indices analysis

**DOI:** 10.1186/s12967-020-02260-9

**Published:** 2020-02-12

**Authors:** Jianying Pei, Yanxia Wang, Yan Li

**Affiliations:** 1 Gansu Provincial Maternity and Child-care Hospital, Lanzhou, 730000 China; 2 Gansu Province Key Laboratory of Biotherapy and Regenerative Medicine, the First Hospital of Lanzhou University, Lanzhou, 730000 China

**Keywords:** Breast cancer, Cancer cell stemness, mRNAsi, WGCNA

## Abstract

**Background:**

With the gradual unveiling of tumour heterogeneity, cancer stem cells (CSCs) are now being considered the initial component of tumour initiation. However, the mechanisms of the growth and maintenance of breast cancer (BRCA) stem cells are still unknown.

**Methods:**

To explore the crucial genes modulating BRCA stemness characteristics, we combined the gene expression value and mRNA expression-based stemness index (mRNAsi) of samples from The Cancer Genome Atlas (TCGA), and the mRNAsi was corrected using the tumour purity (corrected mRNAsi). mRNAsi and corrected mRNAsi were analysed and showed a close relationship with BRCA clinical characteristics, including tumour depth, pathological staging and survival status. Next, weighted gene co-expression network analysis (WGCNA) was applied to distinguish crucial gene modules and key genes. A series of functional analyses and expression validation of key genes were conducted using multiple databases, including Oncomine, Gene Expression Omnibus (GEO) and Gene Expression Profiling Integrative Analysis (GEPIA).

**Results:**

This study found that mRNAsi and corrected mRNAsi scores were higher in BRCA tissues than that in normal tissues, and both of them increased with tumour stage. Higher corrected mRNAsi scores showed worse overall survival outcomes. We screened 3 modules and 32 key genes, and those key genes were found to be strongly correlated with each other. Functional analysis revealed that the key genes were related to cell fate decision events such as the cell cycle, cellular senescence, chromosome segregation and mitotic nuclear division. Among 32 key genes, we identified 12 genes that strongly correlated with BRCA survival.

**Conclusions:**

Thirty-two genes were found to be closely related to BRCA stem cell characteristics; among them, 12 genes showed prognosis-oriented effects in BRCA patients. The most significant signalling pathway related to stemness in BRCA was the cell cycle pathway, which may support new ideas for screening therapeutic targets to inhibit BRCA stem characteristics. These findings may highlight some therapeutic targets for inhibiting BRCA stem cells.

## Background

Breast cancer is one of the most common and lethal cancers in women. According to the latest cancer statistics, the number of estimated new cases and deaths from breast cancer was 268,600 and 41,760, respectively, and the incidence and mortality rates of breast cancer were nearly 30% and 15%, respectively, among all cancers in females worldwide [1]. The incidence rates of breast cancer increased slightly from 2006 to 2015 and this change is considered to be caused by the prevalence of obesity and decrease in parity in women [2]. The most crucial problem in the clinical treatment of breast cancer is that most people first diagnosed with breast cancer are often in an advanced stage because of the lack of access to sensitive markers and effective therapy. Breast cancer is a complex process involving multiple cellular activities and signalling pathways. Hence, it is critical for us to precisely understand the molecular mechanism underlying this complicated malignancy, which could be beneficial for discovering valuable biomarkers to diagnose or predict clinical outcomes.

As a result of the development of single-cell DNA or RNA sequencing technology, tumour heterogeneity is broadly understood and unveils the fact that there are different cell populations in the same tumour tissues, one type of which is cancer stem cells (CSCs) [3, 4]. CSCs show a high degree of plasticity, which leads to distinct cellular phenotypes, functions and metabolic features. One of the reasons for plasticity caused by CSCs is that this cell population has the competence to transform between quiescent and proliferative states when they are stimulated in certain situations [5]. Breast cancer stem cells (BCSCs) were initially reported in 2003 and increasing studies have revealed that BCSCs are closely related to breast carcinogenesis [6]. In addition, the presence of BCSCs was reported to correlate with tumour survival, metastasis and therapy resistance [7]. In addition, progressively increased genes were described to play a role in BCSC regulation in breast tissues, such as metalloproteases (MMPs) and insulin growth factor (IGF), and these genes were upregulated in conventional breast tumour cells [8]. Hence, scientists suspected that cancer cells might arise from a cell population with self-renewal ability, which was thought to be tumour stem cells. Although studies on BCSCs have been continuously conducted worldwide, the role of BCSCs in BRCA pathogenesis and progression is still unclear; identification of the key factors or vital pathways that initiate BCSCs from a quiescent state to a malignant state is urgently needed. To solve these issues, some researchers have used artificial intelligence and deep learning methods to summarize and analyse stem cell features. Malta et al. [9] used a one-class logistic regression (OCLR) machine learning algorithm to extract the transcriptomic and epigenetic feature sets from normal tissue-derived pluripotent stem cells, including embryonic stem cells, induced pluripotent stem cells, and their differentiated progeny, which have different degrees of stemness; in this way, they identified stem cell signatures and quantified stemness with a multi-part analysis containing transcriptomes and methylomes. Finally, two stemness indices, mRNAsi and mDNAsi, were proposed in this study: the former reflected gene expression, and the latter reflected epigenetic features. To verify the two stemness indices, the researchers further annotated and analysed cancer stemness in nearly 12,000 samples of 33 tumour types. Based on this study, we can obtain the stemness indices of each BRCA tissue in the TCGA database.

In the present study, we aimed to recognize key genes and pathways correlated with BRCA stemness by combining mRNAsi in BRCA in TCGA via bioinformatic analysis. The WGCNA model was constructed, and gene modules that are closely related to the mRNAsi index are displayed. We identified three key gene modules and further selected key genes from one of them. Gene and module functional analyses were conducted to show their significance in BRCA. In summary, our study used a novel method to identify stemness-related genes and benefited us by identifying CSC-related genes and predicting their roles in cancer.

## Methods

### Data collection and pre-processing

The RNA sequencing (RNA-Seq) expression data of 1222 samples, including 113 normal samples and 1109 breast cancer samples, and the corresponding clinical information of 1097 cases were downloaded from the TCGA database on September 2019 (https://portal.gdc.cancer.gov). The mRNAsi indices and tumour purity of breast cancer cases in TCGA were obtained from previous studies [9, 13]. We used the Perl language (http://www.perl.org/) to combine the RNA-Seq results of each sample and the Ensemble database (http://asia.ensembl.org/index.html) to convert gene IDs to gene symbols in a matrix profile. After useful information filtering, we took 1097 cases and corresponding clinical information for the next analysis.

### Clinical characteristic correlation analysis

The prognostic value of mRNAsi or corrected mRNAsi was investigated using the survival package in R. The correlation between mRNAsi or corrected mRNAsi and tumour stages or tumour grades was analysed with the beeswarm package in R.

### Screening of differentially expressed genes (DEGs)

Raw expression data from the TCGA were transformed with log2, and identification of differentially expressed genes (DEGs) was conducted using the limma package in R language [10]. The cut-off criteria for DEG selection were as follows: |log2-fold change| > 1, p < 0.01, and false discovery rate (FDR) < 0.05. Volcano plots and heatmaps were drawn using the limma and pheatmap packages in R.

### WGCNA

#### Module establishment

The WGCNA package in R was utilized to build a co-expression network targeting DEGs [11]. All paired genes adopted the average linkage method and Pearson’s correlation matrices, and the co-expression similarity matrix was built using the absolute values of the correlations between transcription data. The function Amn = |Cmn|^β^ (Cmn = Pearson’s correlation between gene m and gene n; Amn = adjacency between gene m and gene n) provided us with a method to establish a weighted adjacency matrix. β defined a correlation power (soft thresholding parameter) showing strong relations between genes and penalizing the weak correlation. We first selected a β value to build a co-expression network, and then we converted the adjacency into a topological overlap matrix (TOM) to measure the network connectivity of genes, and the TOM summed up the adjacent genes for the network gene ratio and calculated the corresponding dissimilarity. We used average linkage hierarchical clustering based on TOM dissimilarity measurement to classify genes showing similar expression profiles with gene modules. The minimum size of the gene group was 30 for the gene dendrogram.

#### Identifying key modules and genes

We chose mRNAsi and epigenetically regulated mRNAsi (EREG-mRNAsi), which is a stemness index generated using a set of stemness-related epigenetically regulated genes, as the sample traits to find CSC-related modules and genes. We selected modules related to the mRNAsi, and genes in these modules were thought to be co-expressed CSC-related genes. First, we calculated the correlation between gene expression levels and sample traits, which was defined as the gene significance (GS). The module eigengenes (MEs) function was used as a key part of the principal component analysis (PCA) for each gene module. In a certain module, the expression model of each gene can be summarized as an expression pattern with a distinct expression feature. The calculation of GS was the log10 transformation of the *p* value (GS = lgp), which reflected a linear regression between the gene expression and mRNAsi or EREG-mRNAsi. Module significance (MS) was the average GS in a specific module, which represented the correlation between the module and sample traits. We merged some quite similar modules using a cut-off (< 0.25), and then the modules that had the largest MS were considered the most sample trait-related modules. After finding the modules of interest, we calculated GS and module membership (MM, correlation between genes in a certain module and gene expression profiles) for each gene. We defined the thresholds for the selection of key genes in a certain module as cor.gene MM > 0.8 and cor.gene GS > 0.5.

### Functional annotation and pathway enrichment analysis

The cluster profiler package in R was selected to perform gene ontology (GO) functional annotation and Kyoto Encyclopedia of Genes and Genomes (KEGG) analyses to investigate and visualize the biological function behind key genes [12]. A *p*-value < 0.05 and an FDR < 0.05 were considered statistically significant.

### Co-expression analysis of key genes and protein–protein interaction (PPI) network analysis

To determine the co-expression relationship between key genes, we chose the corrplot package in R to calculate the Pearson correlations at the transcription level. We used an online tool, Search Tool for the Retrieval of Interacting Genes (STRING), to evaluate the protein–protein interaction (PPI) among key genes.

### Data validation

Oncomine (http://www.oncomine.org) and GEPIA (http://gepia.cancer-pku.cn/) were used to verify the mRNA expression of key genes between tumour and normal tissues in BRCA. The threshold of Oncomine screening was as follows: *p*-value, 1E−4; fold change, 2; gene level, top 10%. We selected three datasets, GSE29431, GSE10797, and GSE65194, from the Gene Expression Omnibus (GEO) database (www.ncbi.nlm.nih.gov/geo/). The online database (http://www.kmplot.com/) was used to examine the survival values of key genes.

### Statistical analyses

All of the cut-offs used in this paper, including mRNAsi, corrected mRNAsi and key gene expression levels, were the median level of each item. The Wilcox test function in R was applied to evaluate the difference in mRNAsi scores or corrected mRNAsi scores between the normal group and the tumour group. A two-sided log-rank test in the survival package in R was employed to assess the survival difference between the two groups. The Kruskal test in R was used to test the correlation between mRNAsi scores or corrected mRNA scores and clinical characteristics. A *p* value < 0.05 was considered statistically significant.

## Results

### mRNAsi and corrected mRNAsi according to clinical characteristics of BRCA

**Fig. 1:**
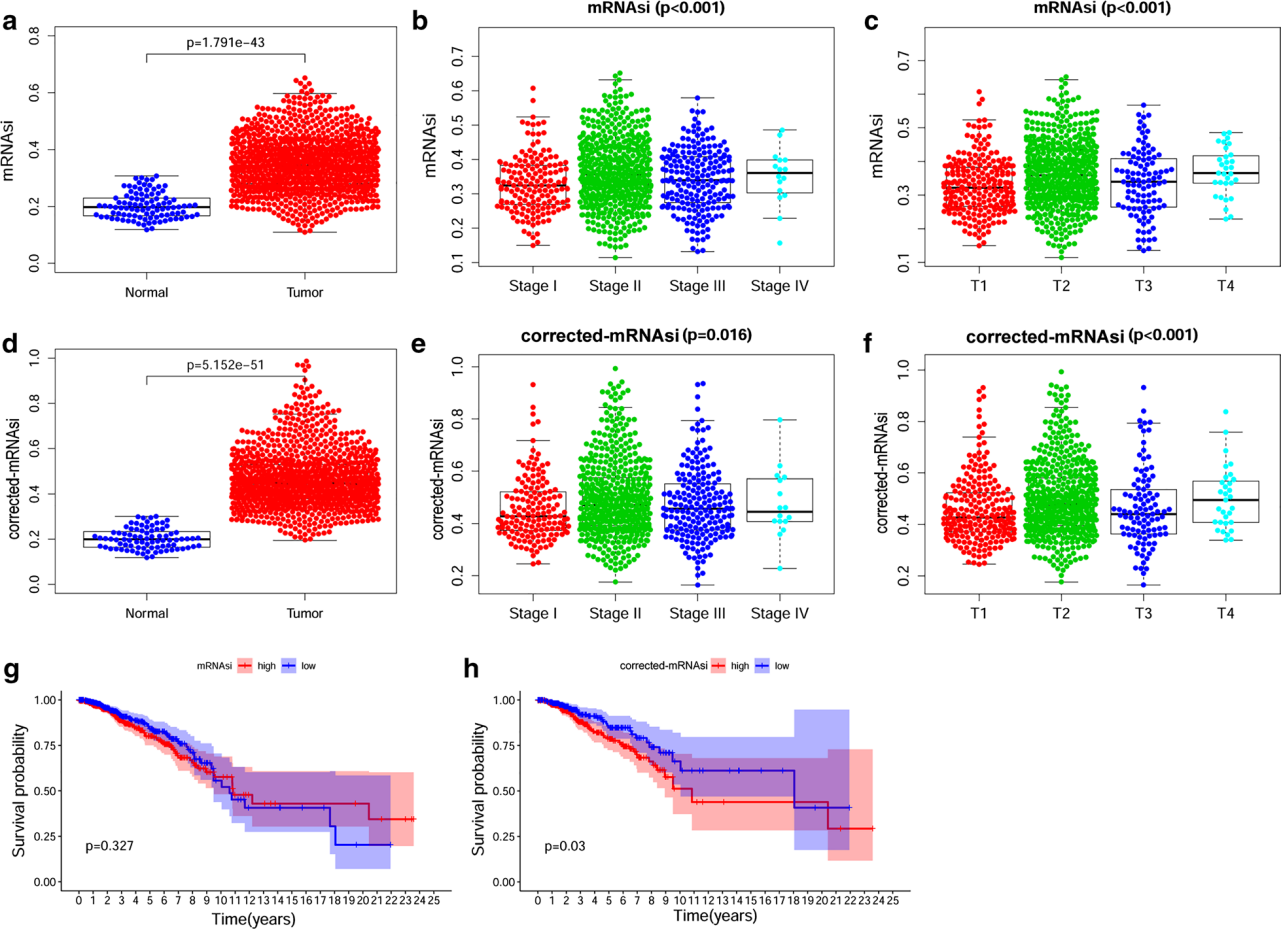
Correlation between mRNAsi/corrected mRNAsi and clinical characteristics in BRCA. **a** Differences in mRNAsi between normal (113 samples) and BRCA (1109 samples) tissues. **b**, **c** Comparison of mRNAsi in different clinical stages (**b**) or tumour depths (**c**) in BRCA. **d** Differences in corrected mRNAsi (mRNAsi/ESTIMATE tumour purity) between normal (113 samples) and BRCA (720 samples) tissues. **e**, **f** Comparison of corrected mRNAsi in different clinical stages (**e**) or tumour depths (**f**) in BRCA. **g**, **h** Kaplan–Meier survival curves of mRNAsi (**g**) or corrected mRNAsi in BRCA (**h**). *p *< 0.05 indicates statistical significance

mRNAsi is a novel stemness index for evaluating the dedifferentiation potential of tumour cells and is thus considered a marker of CSCs. In our study, we found that the mRNAsi in BRCA tissues was significantly higher than that in normal tissues (Fig. 1a). The mRNAsi among different stages of BRCA showed obvious differences, and mRNAsi scores represented a gradually increasing trend with more aggressive clinical traits and tumour stages (Fig. 1b, c). The mRNAsi index was reported to be derived from normal cells and cells with different degrees of stemness via the OCLR algorithm and calculated in TCGA transcriptomic datasets [9]. Tumour tissues were composed of thousands of different cells, including tumour cells and other types of cells, such as stromal and immune cells, which remind us that tumour purity was likely to be an interference factor affecting the evaluation of mRNAsi in clinical characteristics. Given the presence of tumour-associated cells and normal cells in transcriptomic studies, a previous study reported the ESTIMATE method to evaluate tumour purity; thus, the ESTIMATE score of BRCA in TCGA can be obtained [13]. The corrected mRNAsi (mRNAsi/tumour purity), calculated in the same way as a previous report, was used to remedy the influence of tumour purity in our study [14]. We re-analysed the corrected mRNAsi in normal and BRCA tissues and gained a similar result as that with mRNAsi: BRCA tissues had an evidently higher stemness index even after purity correction (Fig. 1d). In addition, the correlation between clinical characteristics and the corrected mRNAsi index was also similar to the correlation with the mRNAsi index (Fig. 1e, f). In the survival analysis, we observed a tendency, without statistical significance, that patients with a higher mRNAsi index had relatively poorer overall survival than those with a lower mRNAsi index (Fig. 1g). Nevertheless, patients with higher corrected mRNAsi values had an apparently poorer survival probability compared with patients who had lower corrected mRNAsi values, and the difference was statistically significant (Fig. 1h). These above results reflected that mRNAsi or corrected mRNAsi in BRCA were closely related to CSCs in terms of clinical characteristics; these results were particularly apparent with the corrected mRNAsi, which may indicate a more accurate relation between CSCs and BRCA.

### DEGs between BRCA tissues and normal tissues

**Fig. 2:**
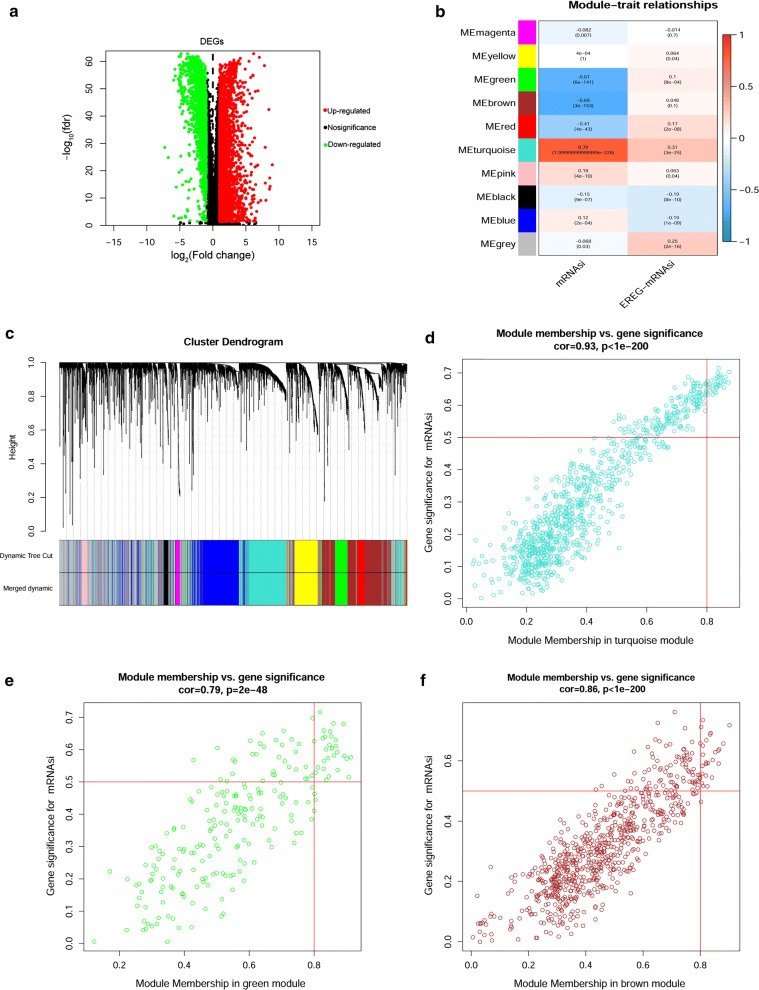
Identification of DEGs and stemness-related key modules in BRCA. **a** Differentially expressed genes: red indicates upregulated genes; green indicates downregulated genes and black indicates genes excluded by DEG screening criteria. **b**, **c** Identification of a co-expression module in BRCA. Each piece of the leaves on the cluster dendrogram corresponded to a gene, and those genes with similar expression patterns compose a branch (**c**). Correlation between gene modules and mRNAsi scores or EREG-mRNAsi. The upper row in each cell indicates the correlation coefficient ranging from − 1 to 1 of the correlation between a certain gene module and mRNAsi or EREG-mRNAsi. The lower row in each cell indicates the *p*-value (**b**). **d**, **e** The scatter plot of the top three important gene modules: turquoise module (**d**), green module (**e**) and brown module (**f**). Each circle indicates a gene, and those circles located in the upper right indicate the key genes in these modules

The above results revealed that mRNAsi scores in BRCA tissues were higher than those in normal tissues; thus, we thought that there were some differentially expressed key genes that regulated the stemness of tumour cells. To identify these genes, we first downloaded the RNA-Seq data from the public TCGA database and screened the DEGs between BRCA tissues and normal tissues. We identified 4575 DEGs, of which 2698 were upregulated and 1877 were downregulated (Fig. 2a and Additional file 1: Table S1).

### WGCNA: identification of mRNAsi-related modules and genes

**Fig. 3:**
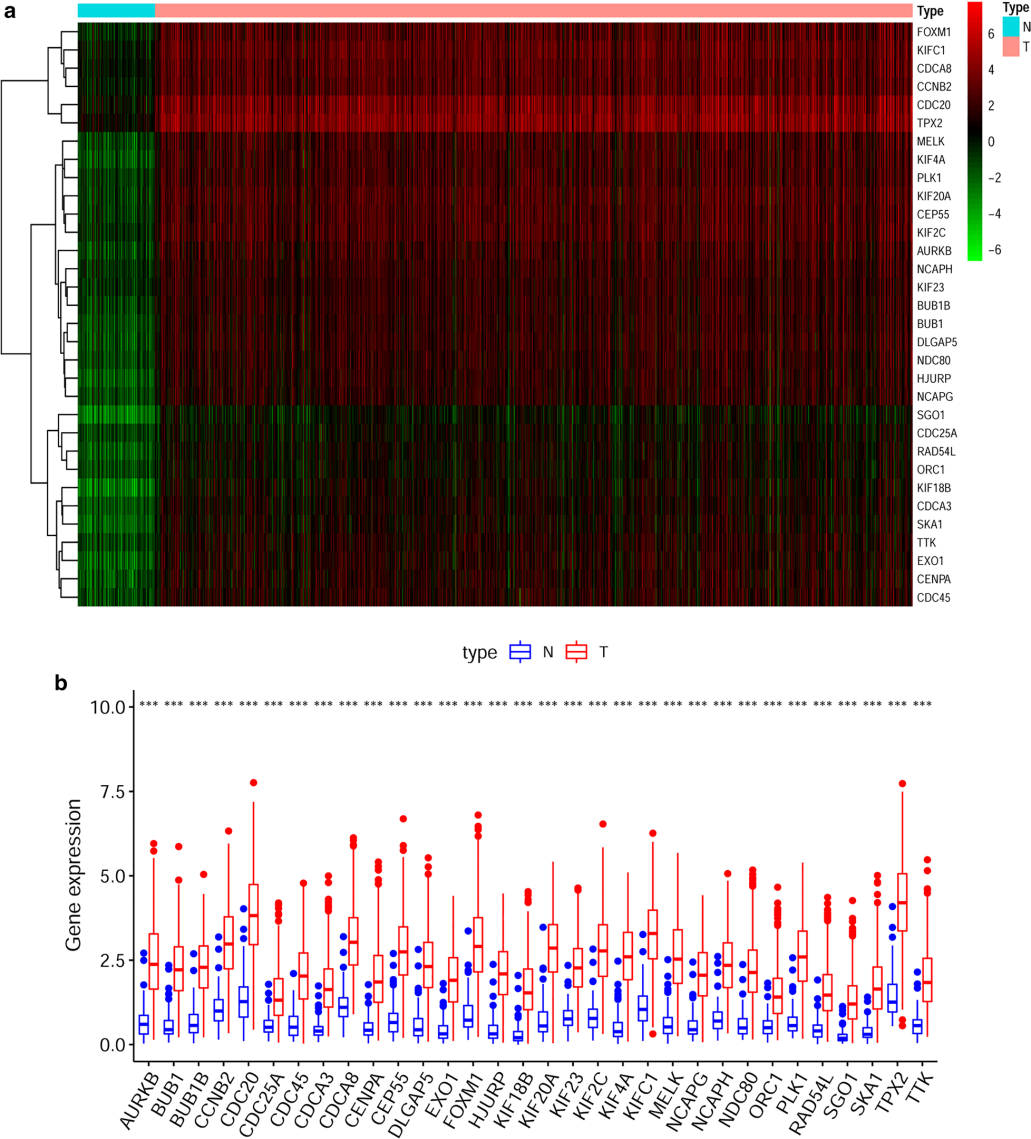
Expression and functional annotation of key genes. **a** Heatmap of the expression level of all key genes. Samples were sorted into normal (N) and tumour (T) groups. Red indicates high expression, and green indicates low expression. **b** The specific expression value of all key genes

WGCNA was applied to construct a DEG co-expression network to classify all DEGs into biological gene modules relying on average linkage hierarchical clustering and to further identify genes strongly associated with BRCA stemness. In this study, we selected β = 7 (scale-free R^2^ = 0.95) as a soft threshold to establish the scale-free network (Additional file 2: Figure S1) and finally obtained 10 gene modules for the next analysis (Fig. 2b, c). To explore the relationship between the gene module and mRNAsi scores, we defined MS as the overall gene expression level of a certain module for the subsequent analysis. As shown in Fig. 2b, the first row of each module was the R^2^ value, which indicated the degree of correlation between gene expression and BRCA stemness; the closer the R^2^ was to 1, the stronger the correlation was. The second row of each module was the *p* value, and *p* < 0.01 was considered statistically significant. Here, we simultaneously analysed the correlation between gene expression and mRNAsi or EREG-mRNAsi. We noticed that there were three modules, including the turquoise module (R^2^ = 0.79, *p *= 8.0e−226) (Fig. 2d), green module (R^2^ = − 0.67, *p *= 6.0e−141) (Fig. 2e) and brown module (R^2^ = − 0.69, *p *= 3.0e−153) (Fig. 2f), which showed very high R^2^ values and quite low *p* values; among these modules, the turquoise module reflected a positive correlation between gene expression and stemness characteristics, while the other two modules showed a negative correlation (Fig. 2b). Then, we selected the turquoise module to screen key genes in the mRNAsi group, and the selection criteria were defined as cor.MM > 0.8 and cor.GS > 0.5. Finally, we screened 32 key genes containing TPX2, HJURP, CDCA8, PLK1, KIFC1, CENPA, CCNB2, KIF2C, EXO1, TTK, KIF4A, CDC25A, MELK, NDC80, NCAPG, CEP55, NCAPH, RAD54L, KIF20A, KIF18B, ORC1, CDC45, KIF23, CDC20, BUB1, AURKB, SKA1, FOXM1, SGO1, DLGAP5, CDCA3, and BUB1B. We extracted the concrete expression value of each key gene and drew the heatmap and box plot, and the results reflected that those key genes were indeed evidently overexpressed in BRCA tissues (Fig. 3a, b).

### Gene function annotation and pathway analysis

**Fig. 4:**
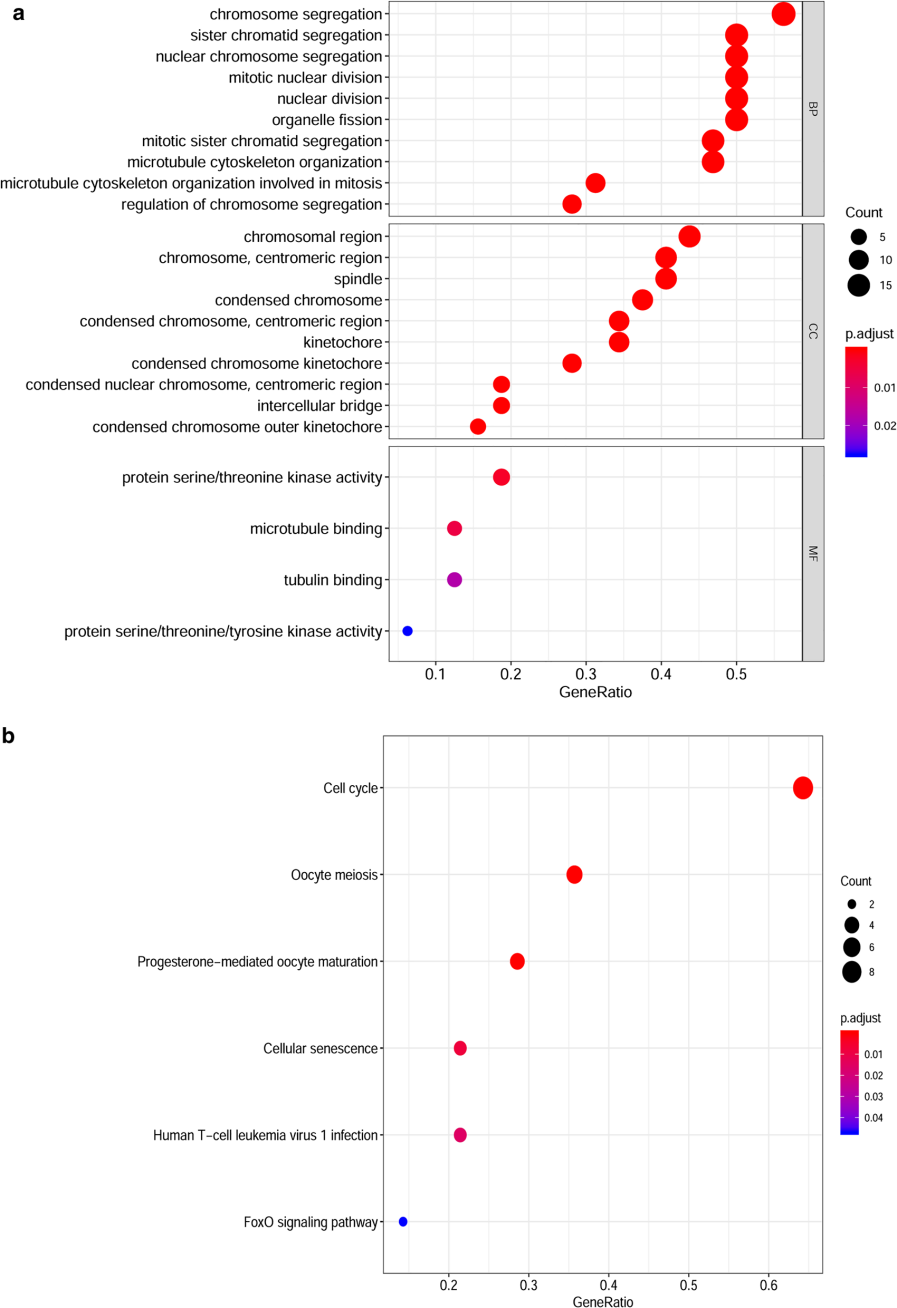
GO and KEGG pathway analysis of key genes

GO and KEGG analyses were employed to perform the functional enrichment analysis of significant modules and key genes. The results revealed that the major biological processes of the turquoise module were organelle fission, nuclear division and chromosome segregation, while the biological functions of the green and brown modules were enriched in extracellular structural organization and ameboid-type cell migration, respectively. In terms of signalling pathway enrichment analysis, we found those modules mainly focused on the cell cycle and PI3K-AKT signalling pathway (Additional file 3: Figure S2). These analyses also showed that the main functions of key genes were chromosome segregation, mitotic nuclear division and microtubule cytoskeleton organization, which are mostly related to the cell cycle pathway (Fig. 4a, b).

### Correlation between key genes at transcription and protein levels

**Fig. 5:**
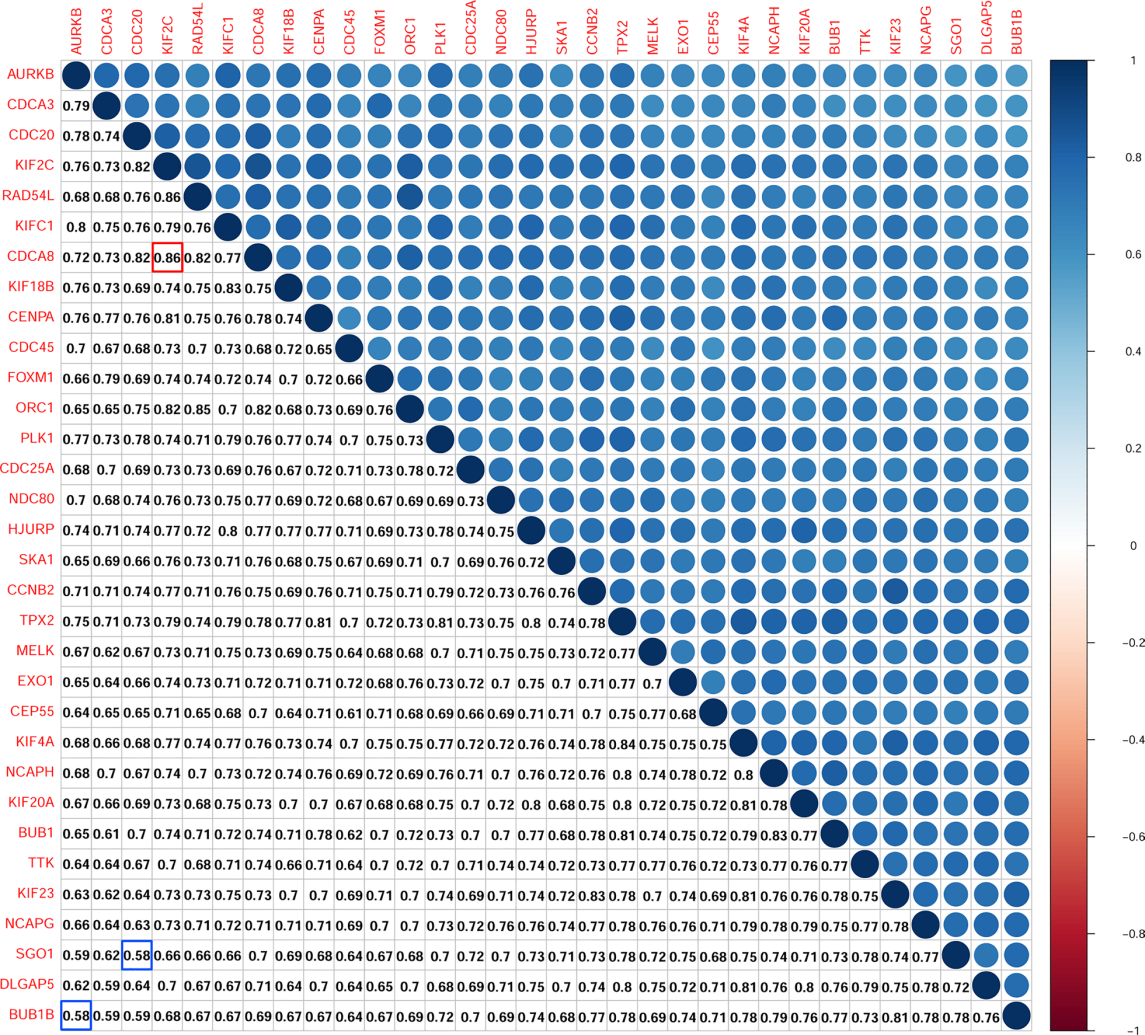
Correlation between key genes at the transcriptional level

**Fig. 6:**
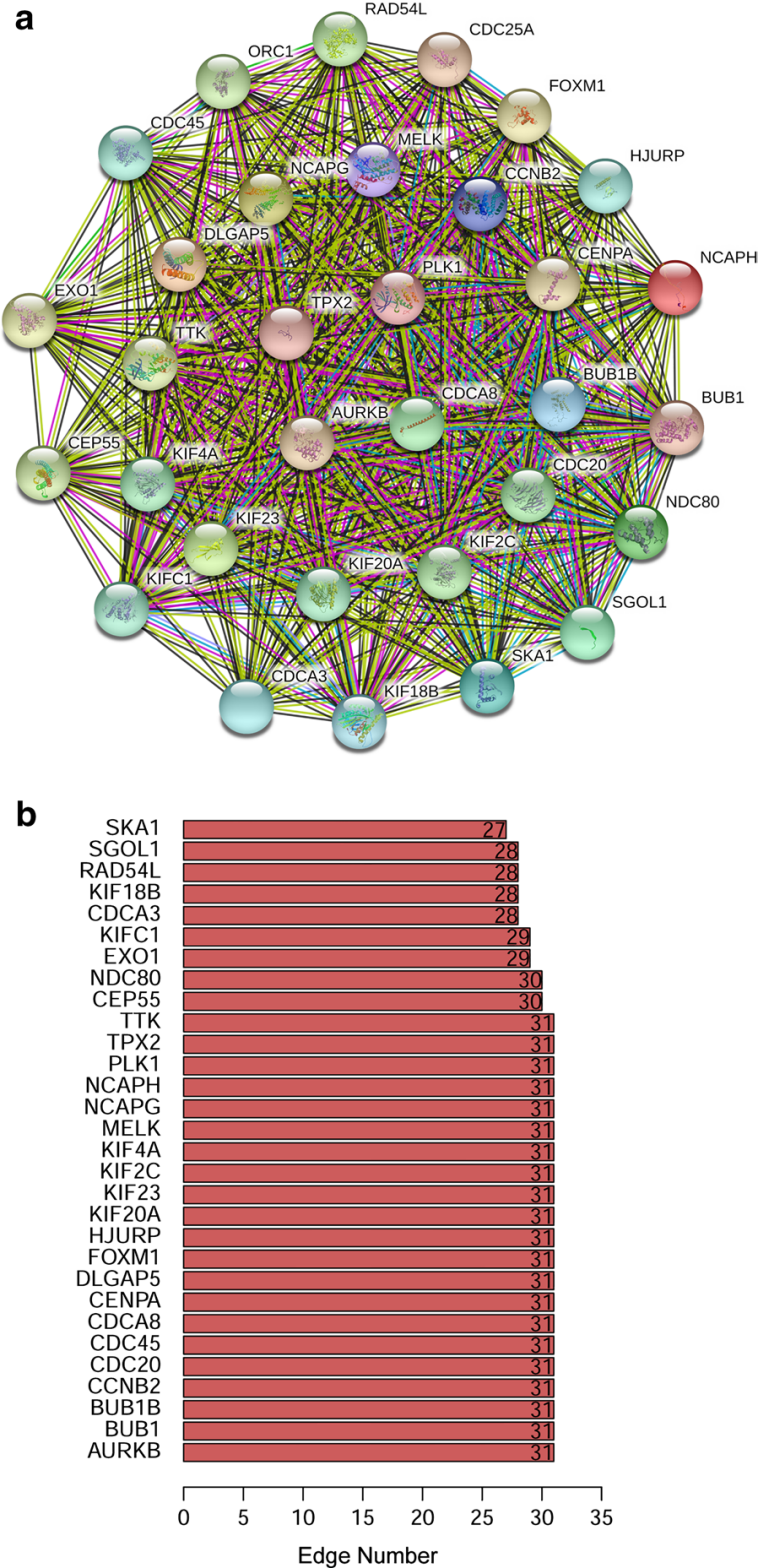
The mutual protein–protein interactions of key genes. **a** and edge number of each key gene (**b**)

We identified the mutual correlation between key genes and their protein products using Pearson correlation and the STRING online tool. As shown in Fig. 5, we found strong and statistically significant correlations between key genes (*p *< 0.01). The relationship between KIF2C and CDCA8 had the highest correlation score of 0.86, and the lowest correlation score of 0.58 was for the relationship between BUB1 and AURKB or SGO1 and CDC20 (Fig. 5). The protein interaction relationships between key genes were analysed using STRING, and a wide-ranging and strong relationship between key genes was shown (Fig. 6a). We analysed the edge number of each node gene in the PPI network, and the results demonstrated an almost equal edge number for each gene, which indicated that those key genes composed a quite dense interaction network (Fig. 6b).

### Validation and analysis of key genes expression

**Fig. 7:**
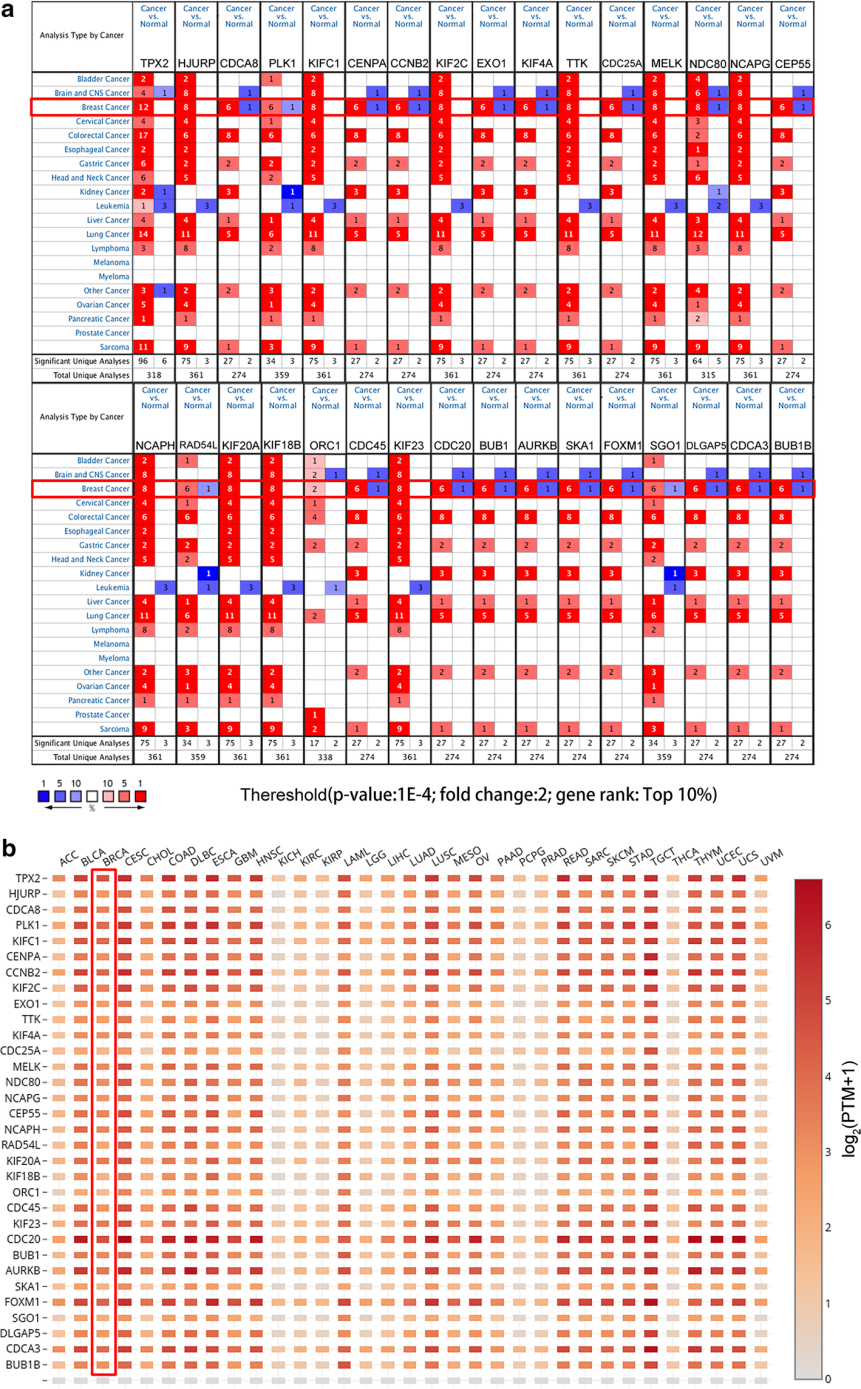
Expression validation of key genes. **a** The mRNA expression of key genes in multiple cancer types from the Oncomine database. The number in the cells represents the number of analyses meeting the thresholds. Red indicates a higher expression level of target genes in tumour tissues than in normal tissues, and blue indicates an opposite expression pattern. The colour depth of each cell indicates the gene rank, and the deeper the colour depth is, the higher the gene rank. **b** The mRNA expression of key genes in multiple cancer types from GEPIA

**Fig. 8:**
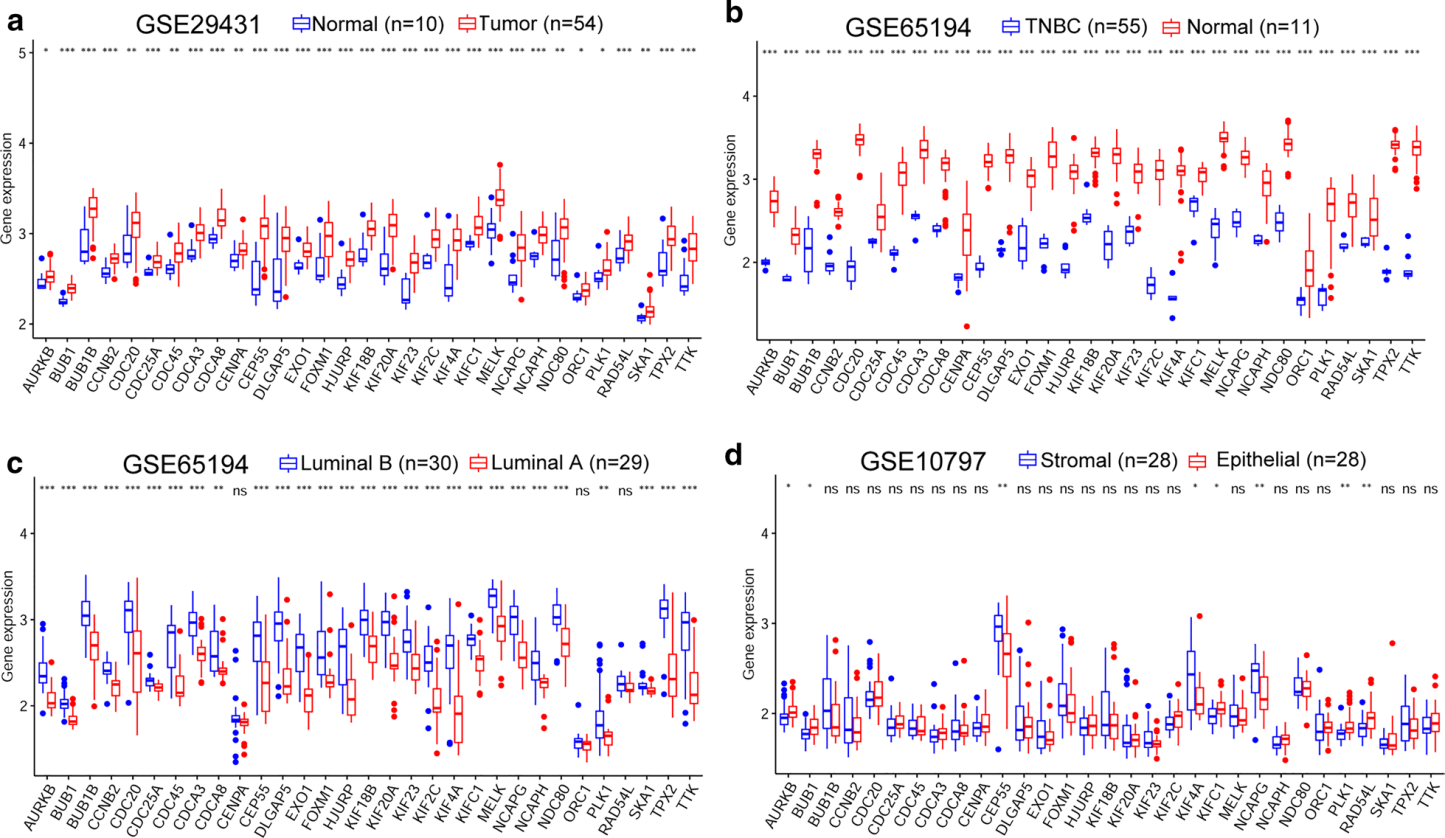
Expression of key genes in the GEO database. The GSE29431 dataset includes normal and BRCA tissues (**a**). The GSE65194 dataset was divided into two pairs: TNBC vs normal tissues (**b**) and luminal B vs luminal A (**c**). GSE10797 includes stromal cells and epithelial cells in BRCA tissues (**d**)

**Fig. 9:**
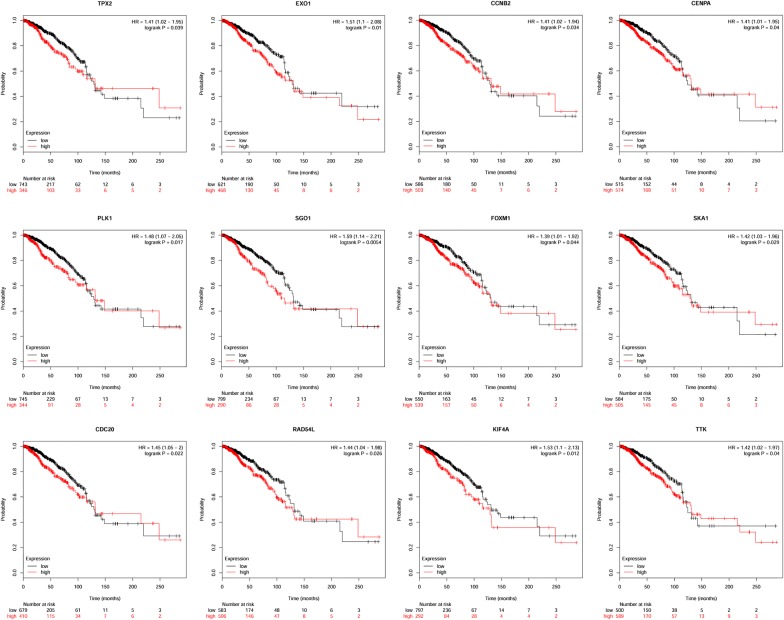
Prognosis-related genes among key genes. Survival analysis of key genes using the Kaplan–Meier plotter online tool

To systematically understand the expression levels of these key genes, we used two databases, Oncomine and GEPIA, to analyse their expression in multiple cancer types. Through the results of analysis using Oncomine, we found that all key genes were obviously upregulated in more than one cancer type in addition to BRCA, and all of them were ranked in the top 10% of DEGs that were ranked with a relatively high number of datasets (Fig. 7a). Meanwhile, we utilized the other online database GEPIA to verify their expression and acquired a similar result, as shown in Fig. 7b. For the expression verification of key genes in BRCA, we chose to examine the GEO data. We first confirmed the expression of key genes between tumour tissues (54 samples) and normal tissues (10 samples) in GSE29431, and all the genes were upregulated (Fig. 8a). Breast cancer is a heterogeneous neoplasm, and distinct subtypes of breast cancer contribute to different clinical outcomes; thus, we evaluated the expression of key genes in different subtypes, including luminal A, luminal B and triple negative breast cancer (TNBC), in BRCA. As shown in Fig. 8b, all 31 key genes (no expression data of SGO1 in GEO) were apparently highly expressed in TNBC compared with normal tissues. Moreover, we found 29 key genes, not including CENPA, ORC1 and RAD54L, that had higher expression levels in luminal B BRCA than that in luminal A BRCA (Fig. 8c). This result demonstrated that these key genes indeed participated in BRCA stemness maintenance. We further analysed the expression of key genes in two cell types containing stromal cells and epithelial cells in BRCA tissues, and the results revealed that only 8 genes (AURKB, BUB1, CENPA, KIF4A, KIFC1, NCAPG, PLK1, and RAD54L) showed distinct expression levels between these two cell types in BRCA tissues (Fig. 8d). To investigate the role of those key genes in BRCA, we performed survival analysis of these genes using the Kaplan–Meier plotter online tool, and the results indicated that 12 genes of the 32 key genes had an effect on the prognosis of patients with BRCA (Fig. 9).

## Discussion

Although many studies have focused on BRCA diagnosis and treatment in recent years, therapeutic strategies to prevent and treat this malignancy are still inadequate and ineffective. With the emergence of the tumour CSC hypothesis, cancer cells are now considered likely to originate from a cell population called stem cells, which has self-renewal capacity; in addition, CSCs have been reported to be involved in tumour progression, therapeutic resistance and recurrence [15]. Thus, it is fairly important and urgent to identify the key genes driving the crucial cellular processes involved in the transformation from quiescent stem cells to non-renewing cancer cells with unlimited proliferative potential. In the present study, we first analysed the correlation between mRNAsi scores and clinical characteristics in BRCA samples and proved that tumour tissues always had higher stemness than normal tissues, which was consistent with previous findings [16]. Considering that the tumour tissues were composed of complex cell types, including cancer-related cells and normal microenvironment cells, and mRNAsi was a stemness index for all cells in a certain sample, to eliminate the bias of mRNAsi caused by non-cancer cells in tumour samples, we calculated corrected mRNAsi scores using the tumour purity. The corrected mRNAsi showed evidently higher levels in BRCA tissues than in normal tissues, and the corrected mRNAsi scores increased as the tumour pathological stage increased, with T4 stage tumours showing the highest stemness. The mRNAsi scores did not show a significant correlation with patient survival unless they were corrected by tumour purity. As previously reported, CSCs mediated tumour metastasis and treatment resistance, which finally predicted poor survival of patients [17].

WGCNA is a tool to analyse the gene expression pattern in multiple samples; it can classify those genes with similar expression patterns into clusters and further analyse the correlations between different gene clusters and certain characteristics [11]. We used WGCNA to initially classify the DEGs into different gene clusters based on a weighted connection analysis of the DEG expression profile between BRCA and normal tissues. Thus, those highly co-expressed genes constituted a gene module that could be used to evaluate the correlation strength between gene modules and the clinical features of interest. In this way, we discovered more than one gene module with strong connections to mRNAsi. Gene function annotation and signalling pathway analysis revealed that these gene modules may exert distinct functions in BRCA; for instance, genes in the green module were most enriched in focal adhesion and ECM-receptor interaction pathways; genes in the brown module were mainly focused on the PI3K-AKT signalling pathway, MAPK pathway and Ras signalling pathway; and genes in the turquoise module were primarily concentrated on the cell cycle and cellular senescence pathways. Given that the cell cycle and cellular senescence determine cell fate and self-renewal, we selected the turquoise module for the next analysis. Based on GS and MM, we selected 32 key genes from the turquoise module. These key genes were all upregulated in BRCA tissues, and gene functional enrichment was most focused on the cell cycle pathway. Some cell cycle regulators have been reported to be involved in not only breast cancer progression but also in the stem-like cell activity of breast cancer cells; for example, inhibition of cyclin D1 or CDK4/6 increases or decreases the migration capacity of stem cells in breast cancer [18]. Additionally, BCSCs are considered to exist in a slow cycling state or a quiescence state, which is the direct consequence of cell cycle dysregulation [19].

The validation of the stemness-related key genes in multiple cancer tissues revealed that most of the key genes were overexpressed in various cancer tissues. In BRCA, the expression levels of these key genes were verified using several GEO datasets, including GSE29431, GSE65194 and GSE10797. As expected, key genes were all overexpressed in BRCA tissues, and the most important was that we found that the expression of key genes in TNBC tissues was quite higher than that in normal tissues. TNBC has a poorer prognosis than other types of breast cancer because of its high degree of malignant phenotypes, which are similar to those of cancer stem cells [20]. We discovered differences in the expression levels of key genes in different subtypes of breast cancer, and 28 of 31 key genes were also upregulated in luminal B tissues compared with luminal A tissues. The expression differences of key genes between TNBC and normal tissues or luminal B and luminal A demonstrated their significance in the regulation of breast cancer stemness characteristics. Only 8 key genes showed expression differences between stromal cells and epithelial cells in breast cancer tissues. We thought the metastasis competence between stromal cells and epithelial cells in the same cancer tissues may not be enough to trigger long-distance metastasis, thus these two cell types may not show evident differences in stemness characteristics. Among all key genes, 12 genes (TPX2, EXO1, CCNB2, CENPA, SGO1, RAD54L, SKA1, FOXM1, PLK1, CDC20, KIF4A and SGO1) were correlated with the survival of BRCA patients.

FOXM1, PLK1, and CENPA composed a cell cycle kinetics regulation pathway in a previous study, which reported that FOXM1 regulated the expression of CENPA and PLK1 to promote mitosis, further regulating the proliferation of pancreatic β cells [21]. The investigation of pancreatic β cells mainly focused on the transition from a quiescent state to a normal cell cycle state, which was quite similar to the characteristics of cancer stem cells. Furthermore, inhibition of PLK1 blocked the growth of CD44 high/CD24-/low tumour-initiating cells in TNBC [22]. CENPA is a critical component of the cell cycle signalling pathway and a necessary regulator of the mitotic spindle; it was found to be expressed in cardiac progenitor cells and to function as a promoter of the proliferation of cardiac progenitor cells [23]. These key genes were mainly focused on the cell cycle signalling pathway, and a previous study reported that cell cycle genes involved in DNA replication and G2 phase progression showed an intrinsic propensity towards the pluripotent state, which suggested that control of the pluripotent state is hardwired to the cell cycle pathway [24].

## Conclusions

In conclusion, 32 genes were found to be closely related to BRCA stem cell characteristics; among them, 12 genes showed prognosis-oriented effects in BRCA patients. The most significant signalling pathway related to stemness in BRCA was the cell cycle pathway, which may support new ideas in therapeutic target screening for inhibitors of BRCA stem characteristics. Conclusions derived from bioinformatic analysis of retrospective data certainly need to be validated by further biological studies, and this is what we are going to do next.

## Supplementary information

**Additional file 1: Table S1.** The DEGs between BRCA and normal tissues.

**Additional file 2: Figure S1.** (A) Clustering of samples and removal of outliers. (B) Analysis of topology for thresholding powers in scale independence and mean connectivity.

**Additional file 3: Figure S2.** GO and KEGG analysis of three modules of interest (green module, brown module and turquoise module).

## Data Availability

The datasets supporting conclusions of this article are available in the TCGA (https://portal.gdc.cancer.gov), Oncomine (http://www.oncomine.org), GEPIA (http://gepia.cancer-pku.cn/), GEO (www.ncbi.nlm.nih.gov/geo/). The datasets supporting the conclusions of this article are included within the article and its Additional files.

## Abbreviations

CSCs: Cancer stem cells
BRCA: Breast cancer
mRNAsi: mRNA expression-based stemness index
EREG-mRNAsi: Epigenetically regulated mRNAsi
TCGA: The Cancer Genome Atlas
WGCNA: Weighted gene co-expression network analysis
GEO: Gene Expression Omnibus
GEPIA: Gene Expression Profiling Integrative Analysis
BCSCs: Breast cancer stem cells
MMPs: Metalloproteases
IGF: Insulin growth factor
OCLR: One-class logistic regression
TNBC: Triple negative breast cancer
DEGs: Differentially expressed genes
FDR: False discovery rate
TOM: Topological overlap matrix
GS: Gene significance
MEs: Module eigengenes
PCA: Principal component analysis
MM: Module membership
GO: Gene ontology
KEGG: Kyoto Encyclopedia of Genes and Genomes
STRING: Search Tool for the Retrieval of Interacting Genes
PPI: Protein–protein interaction
