# Supplementary figures and images for: Identification of key genes controlling breast cancer stem cell characteristics via stemness indices analysis
Source: J Transl Med. 2020 Feb 12;18:74. doi: 10.1186/s12967-020-02260-9 (PMC7014665; doi:10.1186/s12967-020-02260-9)

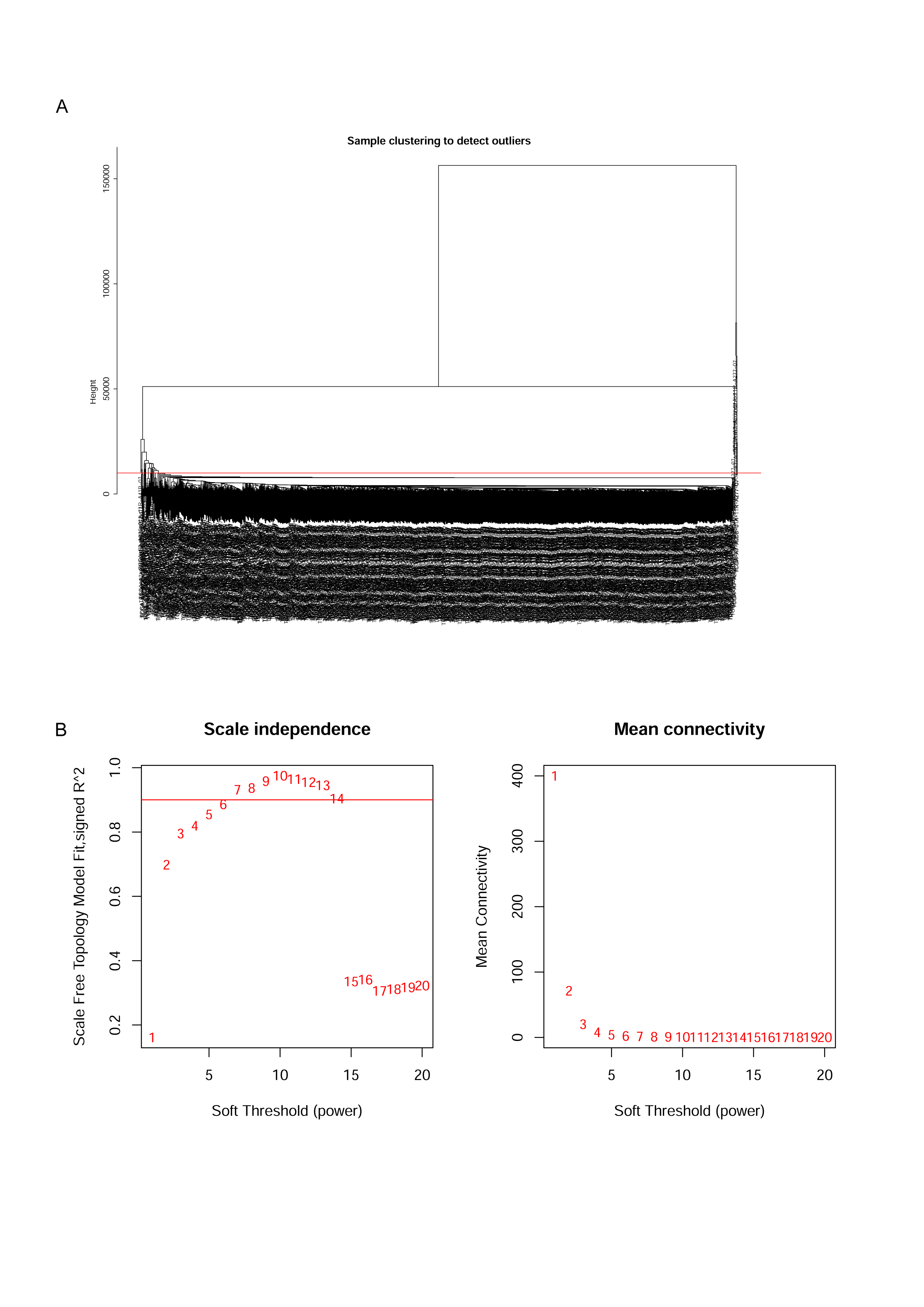

Supplement: Supplementary file 2 — Additional file 2: Figure S1. (A) Clustering of samples and removal of outliers. (B) Analysis of topology for thresholding powers in scale independence and mean connectivity. [file 12967_2020_2260_MOESM2_ESM.tif]
